# Unwitnessed Foreign Body Ingestion Causing Significant Morbidity in a Pediatric Patient Who Died During Resuscitation Secondary to Acute Upper Gastrointestinal Bleeding

**DOI:** 10.7759/cureus.38752

**Published:** 2023-05-09

**Authors:** Haitham Almoffarreh, Omar Alawni, Ahmad Mustafa, Azzam Aljaafari

**Affiliations:** 1 Pediatric Emergency Medicine, King Abdullah Specialist Children’s Hospital, King Abdulaziz Medical City, Riyadh, SAU; 2 Pediatric Gastroenterology, King Abdullah Specialist Children’s Hospital, King Abdulaziz Medical City, Riyadh, SAU

**Keywords:** pediatric, morbidity, esophageal perforation, upper gastrointestinal bleeding, foreign body ingestion

## Abstract

Foreign body (FB) ingestion is a common presenting complaint to the emergency department in the pediatric age group; however, management and intervention vary based on the object ingested, location, time since ingestion, and clinical presentation. One of the rare presentations of foreign body ingestion is extreme complications such as upper gastrointestinal (GI) bleeding that requires urgent resuscitation and might need surgical intervention. We urge critical healthcare providers to consider foreign body ingestion in the differential diagnosis of acute unexplained upper gastrointestinal bleeding and maintain a high index of suspicion, and they must endeavor to obtain a complete history.

## Introduction

Foreign body ingestion is one of the commonest presentations to the emergency department, with peak prevalence between six months and six years of age [1-4]. Most foreign body ingestions in children are accidental and found in the home environment, such as toys, coins, batteries, magnets, and other sharp objects [5]. The majority of the foreign bodies pass spontaneously through the gastrointestinal (GI) tract. However, serious complications such as upper gastrointestinal bleeding, esophageal perforation or laceration, complete esophageal obstruction, aortoesophageal fistula (AEF), and bowel perforation can occur [5,6].

## Case presentation

A 25-month-old female, medically and surgically free, vaccinated up to 18 months of age, presented acutely to the emergency department with fresh blood vomiting with volume around 40- 60 mL, preceded with a history of hypoactivity and melena for the past two days. The family denied any history of cough or pain, fever, bruising, petechiae, or blood oozing from any other site.

The family took the patient to a private clinic where she was found to have low hemoglobin (6 g/dL), and the patient was urgently transferred to the nearest tertiary center. Physical examination upon receiving the patient in our facility revealed an awake child but pale and hypoactive. Vitals included a temperature of 37.5°C, heart rate between 130 and 150 beats/minute, systolic blood pressure borderline trending down from the 90s to 70s mmHg, diastolic blood pressure down from 45s to 35s mmHg, capillary refill time of 3-4 seconds, respiratory rate of 25 breaths/minute, and oxygen saturation of 98% on room air.

There were no signs of bleeding from any other site, no petechiae, or bruises. Oral cavity, abdominal, chest, cardiovascular, and central nervous system examinations were normal.

**Figure 1:**
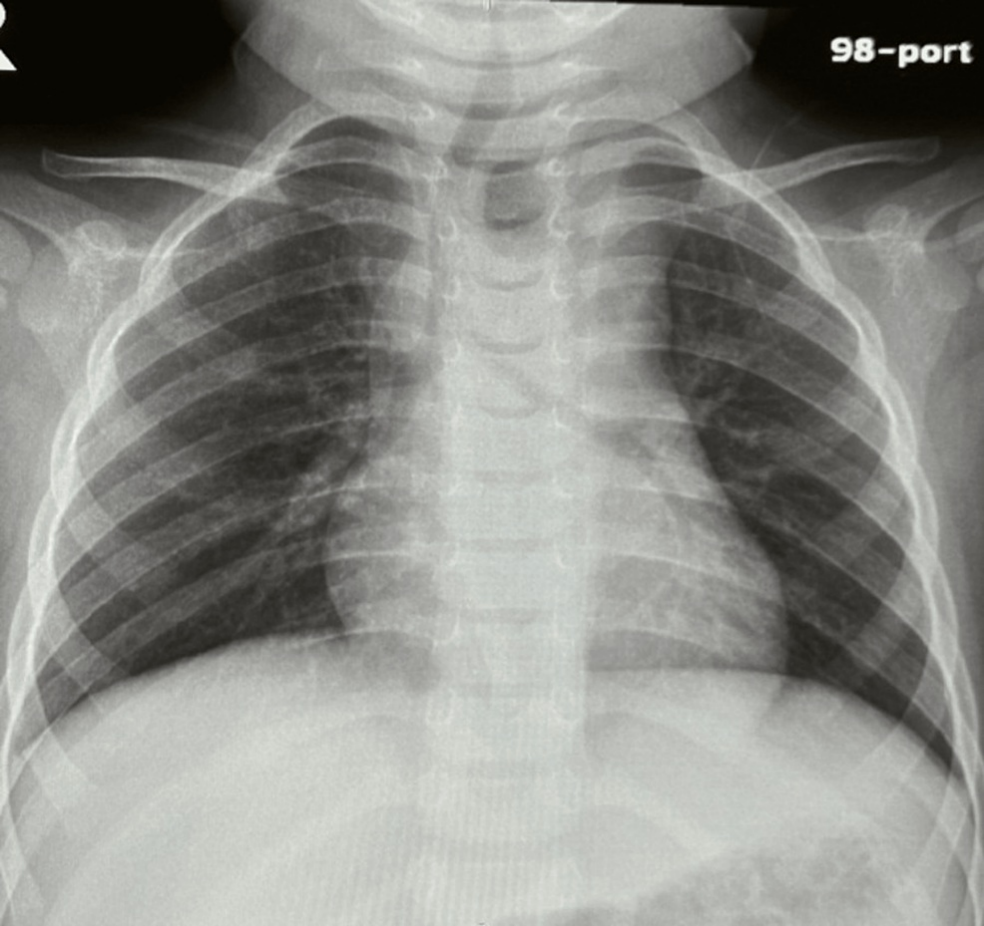
Chest X-ray showing a rounded lucency in the upper esophagus, pushing the trachea to the opposite side with the widening of the mediastinum

The patient vomited two times fresh blood in the resuscitation area. She was given fluids, 20 mL/kg intravenous (IV) bolus 0.9% sodium chloride with intravenous (IV) dextrose 5% normal saline, was kept nil per os (NPO), received granisetron as an antiemetic, omeprazole, prophylactic antibiotic, and was transfused O-ve blood. A chest X-ray showed a rounded lucency in the upper esophagus, pushing the trachea to the opposite side with the widening of the mediastinum (Figure 1). So, the decision was made to do computed tomography (CT) after stabilization.

After initial stabilization in the emergency department, blood workup showed hemoglobin of 4 g/dL, white blood cell counts of 11.6 × 10^9^ per liter (neutrophils: 64.3%, lymphocytes: 3.24%), and platelets of 238 × 10^9^ per liter. Coagulation study, biochemistry, and liver function tests are all within the normal range.

An endotracheal tube (ETT) secured the patient’s airway, and then, she underwent computed tomography (CT), which showed a rounded high-density structure seen at the posterior mediastinum at the level of thoracic 2 to thoracic 4, measuring 1.8 cm, representing ingestion of foreign body, which is associated with air pocket adjacent to the left upper esophagus at the left upper mediastinum, suggestive of esophageal perforation and a posterior mediastinal mass measuring about 2.8 × 4.8 cm in axial diameter. Heterogenous density is noted adjacent to the aortic arch, likely representing hematoma causing mass effect and displacing the trachea and upper esophagus anteriorly and causing indentation on the proximal right main bronchus. Abdominal CT showed dilated lower esophagus, filled and distended stomach, and hyperdense materials, likely bloody products; otherwise, it was unremarkable.

**Figure 2:**
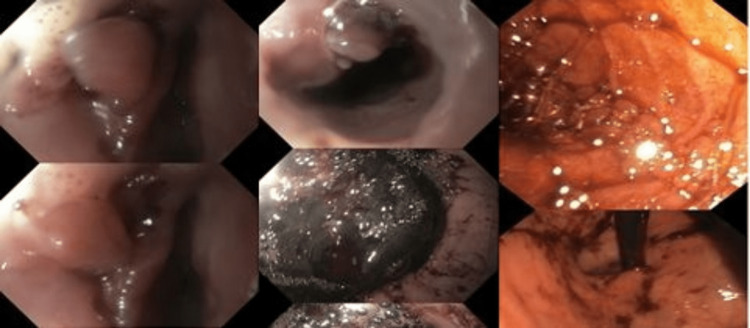
Upper GI scope showing esophageal diverticula and cystic-like lesion in the upper esophagus GI: gastrointestinal

**Figure 3:**
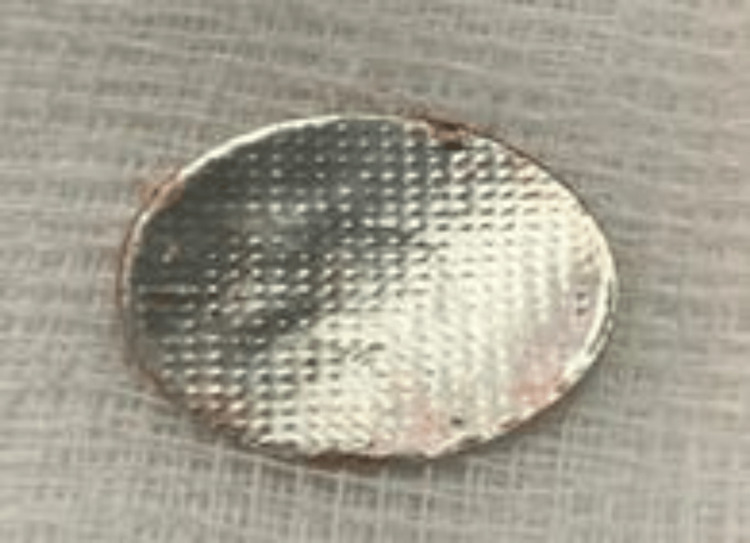
Metallic rounded foreign body

Pediatric intensivist (pediatric intensive care unit (PICU)), pediatric surgery, and pediatric gastroenterology teams were consulted, and the patient was rushed to the operation room (OR) for emergency diagnostic and therapeutic exploration. In the OR, the patient underwent bilateral thoracotomy, and endoscopy was attempted by the gastrology team, which found esophageal diverticula and cystic lesion, bypassed by a guide wire and showed a metallic rounded foreign body, which was removed successfully (Figure 2 and Figure 3).

The patient had an uneventful postoperative period and was hospitalized in the pediatric intensive care unit (PICU) before being transferred to the general ward. She stayed in the hospital for three weeks and was discharged safely with normal vital signs and blood profile. However, three days after discharge, the patient presented with life-threatening upper gastrointestinal bleeding. Despite being resuscitated in the emergency department, the patient did not achieve a return of spontaneous circulation (ROSC) and was declared deceased at that time.

## Discussion

Foreign body ingestion is frequently seen in the emergency department. Most foreign body ingestion occurs below three years of age, with most of these foreign bodies being coin [7]. Patients can present with dysphagia, vomiting, drooling, feeding refusal, and neck and chest pain. The nature of the complication depends on the type, time, and location of the foreign body. The longer the duration of impaction with a sharper foreign body, the higher the risk of complication [8].

Aortoesophageal fistula is a rare cause of pediatric upper gastrointestinal tract bleeding with high mortality because of massive bleeding. A foreign body is one of the commonest causes of aortoesophageal fistula in pediatric patients, mostly as part of button battery ingestion. Sharp objects may perforate the wall directly or with pressure necrosis causing chronic inflammation, which can lead to fistula development [1]. The bleeding can occur after a period following foreign body removal.

In our case, the foreign body was a metallic rounded object unwitnessed by the family. The chest X-ray did not identify it, and it was not initially seen by a regular endoscope, so a guide wire was needed to identify it. It stayed for a long time, forming granulation tissue and a fistula.

We recommend considering undergoing CT angiography to evaluate for aortoesophageal fistula if chronic foreign body ingestion is suspected as the risk of aortoesophageal fistula still exists.

## Conclusions

Upper gastrointestinal bleeding is a life-threatening condition that can lead to severe complications and mortality. We emphasize that healthcare providers and emergency physicians keep foreign body ingestion as one of the top differential diagnoses that can lead to upper gastrointestinal bleeding, especially in the pediatric age group, as they can ingest foreign body materials without family awareness or witness. A patient who presents similarly to our case report should be followed closely and frequently after being discharged home due to the risk of aortoesophageal fistula, which can lead to massive uncontrolled bleeding, ultimately causing loss of life.
